# Post-marketing Surveillance of Intranasal Nalmefene: Prevalence and Clinical Relevance of Precipitated Withdrawal

**DOI:** 10.7759/cureus.95174

**Published:** 2025-10-22

**Authors:** Jordan Paavola, Phil Skolnick, Ann Wheeler, Baher Mankabady, Celine M Laffont, Christian Heidbreder

**Affiliations:** 1 Medical Affairs and Safety, Indivior, Inc., North Chesterfield, USA; 2 Research and Development, Indivior, Inc., North Chesterfield, USA

**Keywords:** fentanyl, nalmefene, opioid overdose, precipitated withdrawal, synthetic opioids

## Abstract

Background: Intranasal (IN) nalmefene (OPVEE®) was approved as an opioid overdose reversal agent with a pharmacological profile distinct from IN naloxone, raising early concerns about the potential for precipitated withdrawal despite the absence of evidence.

Objective: This study aimed to evaluate the prevalence and clinical significance of precipitated withdrawal following the administration of IN nalmefene using post-marketing surveillance data.

Methods: We conducted a comprehensive analysis of adverse event reports submitted to Indivior's Global Safety Database following the commercial launch of IN nalmefene in October 2023. Reports were screened for terms consistent with opioid withdrawal syndromes, and cases were assessed for temporal association, severity, and clinical outcomes.

Results: Through July 2025, there have been 246 confirmed reversals with IN nalmefene across the United States. Among the total adverse event reports received (n=15), instances of precipitated withdrawal were rare (n=2). Most reports lacked sufficient clinical details to confirm causality, and no consistent pattern of severe or treatment-limiting withdrawal emerged. The overall signal strength for precipitated withdrawal was low, with no indication of a disproportionate reporting trend.

Conclusions: Post-marketing surveillance data suggest that the risk of precipitated withdrawal with IN nalmefene is low in real-world settings. These findings support the continued deployment of IN nalmefene as a life-saving intervention while underscoring the importance of ongoing pharmacovigilance to monitor for emerging safety signals.

## Introduction

Despite ongoing efforts to combat the opioid crisis, synthetic opioids like fentanyl continue to drive alarmingly high rates of fatal and nonfatal overdoses in the United States. The Centers for Disease Control and Prevention (CDC) reported 50,900 fatal opioid overdoses in the 12-month period ending February 2025, with almost 90% linked to synthetic opioids like fentanyl [1]. The severity of this crisis is underscored by a report from the Office of National Drug Control Policy (ONDCP) estimating between five and 14 nonfatal overdoses for every fatal opioid overdose reported [2].

Both intranasal (IN) and intramuscular (IM) naloxone are widely used as opioid overdose reversal agents in the community setting [3]. While naloxone is a well-established opioid receptor antagonist [4], accumulating evidence indicates that higher doses of naloxone are often necessary to reverse overdoses involving synthetic opioids compared to those caused by opium-based alkaloids [5-10]. Findings from clinical pharmacology studies [10] and a validated translational model of synthetic opioid overdose [11,12] show that "standard" doses of naloxone (i.e., 2 mg IM, 4 mg IN) used in a community setting could result in preventable fatalities when confronted with a potentially lethal dose of a synthetic opioid such as fentanyl. 

Nalmefene is a competitive µ-opioid receptor (MOR) antagonist with a higher affinity (~5-fold) [13] and a substantially longer (~11 h) plasma half-life [14,15] than naloxone. The Food and Drug Administration (FDA)-approved formulation of IN nalmefene (OPVEE®, currently manufactured by Indivior) delivers higher plasma concentrations than 4 mg IN naloxone [14,15] in the first five minutes and produces a more rapid reversal of opioid-induced respiratory depression [16]. Consistent with these pharmacological properties, simulations using the FDA's translational model of synthetic opioid overdose [11,12] demonstrated that a single dose of IN nalmefene produced large and clinical meaningful reductions in the incidence of cardiac arrest (a surrogate measure of death in the absence of intervention [17]) compared to a standard dose of either IM (2 mg) or IN (4 mg) naloxone [18,19].

The rapid delivery of high concentrations of a high-affinity opioid receptor antagonist, combined with a long plasma half-life, raises the specter of profound and protracted withdrawal symptoms in patients with an opioid use disorder (OUD). These concerns have been voiced in the recent literature [20-22]. 

Here, we detail post-marketing surveillance data for IN nalmefene available through July 2025 to evaluate the prevalence and clinical significance of precipitated withdrawal in real-world settings.

## Materials and methods

Confirmed rescues with IN nalmefene were collected through voluntary reports submitted to Indivior and were internally documented based on the state in which the rescue occurred. Indivior's Global Safety Database, maintained at corporate headquarters in Richmond, Virginia, United States, was searched for cases of precipitated withdrawal involving IN nalmefene received between October 2023 (commercial launch) and July 2025. Cases were identified using the Medical Dictionary for Regulatory Activities (MedDRA) [23] standard MedDRA query (SMQ) for drug withdrawal. Safety data were collected and analyzed in accordance with international pharmacovigilance standards, including the International Council for Harmonisation (ICH) Harmonised Tripartite Guideline: Pharmacovigilance Planning E2E and applicable regulatory guidance [24]. Adverse events and serious adverse events were coded using MedDRA Version 28.0 and assessed for seriousness, expectedness, and causality using the Bradford Hill criteria [25]. These criteria include temporality (exposure precedes outcome), strength and consistency of association across cases, specificity of the exposure-effect link, presence of a biological gradient, and alignment with established biological mechanisms (plausibility and coherence). While not all criteria must be met for medical evaluation, their collective evaluation strengthens causal interpretation. Data sources included spontaneous reports, clinical trial safety databases, literature surveillance, and post-marketing surveillance systems. Signal detection is typically conducted using both quantitative disproportionality analyses (e.g., proportional reporting ratio (PRR) to look for signals of disproportionate reporting (SDR)) and qualitative clinical review. However, due to the limited number of cases, according to FDA Good Pharmacovigilance Practices and Pharmacoepidemiologic Assessment guidance, quantitative disproportionality analyses were not applicable; therefore, only qualitative review was conducted. Identified signals were further evaluated through case series analysis and medical review to determine clinical relevance and potential risk. All findings were documented in accordance with Good Pharmacovigilance Practices (GVP) and subject to internal quality control procedures.

## Results

Adverse event reports

Through July 2025, 246 confirmed rescues with IN nalmefene were reported across the United States. This figure is based on voluntary reports submitted to Indivior and may underestimate the true number of opioid overdose reversals. Fifteen spontaneous cases of adverse events involving the use of IN nalmefene were reported over the analysis period, with no solicited reports or reports from the literature describing adverse events. Among these 15 cases, four reflected qualitative reports of an adverse experience in general (i.e., not pertaining to a specific patient), and 11 cases reflected individual patient experience. Of the 11 patient reports, two patients (4-8 per 1,000 doses used, assuming 1-2 doses per rescue) experienced symptoms suggestive of precipitated withdrawal. The following describes the chronology of events for these two patients.

Case 1

A registered nurse described a male patient of unknown age who had arrived at a correctional facility around 2:00 AM with active emesis, indicative of drug withdrawal. At 8:00 AM, the patient was administered a single 2.7 mg dose of IN nalmefene. Administration of IN nalmefene was considered off label as there were no reported clinical signs suggestive of either central nervous system depression or respiratory depression and the patient's symptoms were not consistent with an acute overdose presentation. Post-administration, the patient exhibited persistent vomiting and tremors, necessitating hospital transfer. He was diagnosed with aspiration pneumonia and precipitated withdrawal. Both conditions resolved, and the patient was subsequently discharged. The event was considered related to IN nalmefene.

Case 2

A non-healthcare professional reported a case involving a male patient of unknown age who experienced precipitated withdrawal after receiving a 2.7 mg dose of IN nalmefene. The patient had just taken his routine methadone dose when he began to "nod off" during an assessment. Suspecting an overdose, a nurse administered IN nalmefene, its first use at the facility, resulting in unspecified withdrawal symptoms. The outcome remains unknown, and no further follow-up was permitted. The event was considered related to IN nalmefene. 

Data from Michigan

About 35% of the reported reversals with IN nalmefene have occurred in Michigan. As a result, post-marketing surveillance data from this state were evaluated to assess safety concerns, particularly the incidence of precipitated withdrawal. To date, there have been no precipitated withdrawal adverse events reported from Michigan. The absence of reported precipitated withdrawal in this mixed urban and suburban setting suggests a favorable safety profile, especially considering the scrutiny around the risk of precipitated withdrawal for this product. 

Case report

These post-marketing surveillance data are consistent with a recently published case report describing the independent review by two clinical experts of body camera footage of a first responder administering IN nalmefene to an individual with a suspected opioid overdose. They documented an increase in oxygen saturation up to 98% within approximately 2.5 minutes, a marked improvement in mental status, and no evidence of precipitated withdrawal [26]. 

Post-marketing requirement

A condition of FDA approval was enhanced pharmacovigilance, requiring 15-day "alert reports" for all cases of severe, prolonged, or precipitated opioid withdrawal involving more than two doses of IN nalmefene during a single rescue, through five years post-approval. These events must also be analyzed separately in quarterly (years 1-3) and annual (years 4-5) post-marketing safety reports, with cumulative assessments of risk factors, outcomes, and causality. As of July 2025, there have been no cases reported which meet these criteria. 

## Discussion

Precipitated withdrawal is the result of an abrupt reduction in the occupation of MORs by an agonist (e.g., fentanyl) caused by displacement with either an antagonist (naloxone, nalmefene) or partial agonist (buprenorphine) in individuals who are physically dependent on opioids [8]. Precipitated withdrawal is therefore a class effect of all currently approved opioid overdose reversal agents, with its likelihood and severity depending on the concentration, affinity, and lipophilicity of both opioid agonist and antagonist, the efficacy of the opioid agonist, and the functional status of MORs that appears correlated with the degree of opioid tolerance [27]. The high affinity of nalmefene for MORs [13], combined with the rapid attainment of elevated plasma concentrations within the first five minutes following IN administration [15,16], suggests that if precipitated withdrawal were to occur, symptoms would likely manifest more rapidly after a single dose of IN nalmefene than after a single 4 mg IN dose of naloxone commonly used in community settings.

Despite concerns that IN nalmefene could precipitate severe opioid withdrawal, pharmacovigilance data collected to date do not indicate any such signal in real-world settings. It is important to recognize, however, that the opioid landscape is rapidly evolving. The increasing prevalence of synthetic opioids such as fentanyl, often associated with the development of high levels of opioid tolerance [28], may influence both the likelihood and severity of precipitated withdrawal events.

Synthetic opioids are orders of magnitude [29,30] more lipophilic than opium-based molecules such as morphine resulting in both rapid brain penetration and the onset of pharmacological actions, including respiratory depression [5,31,32]. For fentanyl, a well-studied synthetic opioid, the rapid uptake into the central nervous system is followed by a redistribution to peripheral tissues including muscle and adipose [33]. Converging lines of evidence indicate that this redistribution to peripheral tissues creates a reservoir [33], slowly releasing fentanyl back into the circulation. For example, patients entering OUD treatment tested positive for fentanyl or its metabolite (norfentanyl) in urine for 7-13 days (with one participant testing positive for norfentanyl for 26 days) compared to 2-4 days typically seen with short-acting opioids [34]. This sustained fentanyl exposure in chronic users could mitigate the manifestation of severe precipitated withdrawal when a reversal agent is administered, by maintaining sufficient activity at the MORs. However, this hypothesis does not explain the higher incidence of precipitated withdrawal observed with buprenorphine initiation in OUD patients using fentanyl compared to those not using fentanyl [35,36]. Unlike opioid antagonists like naloxone and nalmefene, buprenorphine dissociates extremely slowly from MORs (dissociation rates are ≥20-fold lower than naloxone and nalmefene [13]), producing a durable block that prevents activation by opioid agonists, including fentanyl [37,38]. Clearly, additional studies are needed to better understand the factors contributing to the development of precipitated withdrawal, including the role of adulterants commonly found in the illicit drug supply (benzodiazepines and alpha-2 agonists [39]), which may complicate withdrawal symptoms.

Limitations

Despite the robustness and regulatory oversight of post-marketing surveillance in the pharmaceutical industry, there are limitations inherent to the self-reported (prescriber/patient) nature and lack of standardization of pharmacovigilance data. However, if major safety issues were to arise, they would be captured by the pharmacovigilance system. Another limitation is that some individuals who experience an opioid overdose report not intending to take opioids [40]. This raises the potential of an overdose in non-tolerant individuals who will not exhibit the same intense withdrawal symptoms following overdose reversal. Finally, due to the small sample size, these findings should be considered preliminary and will require confirmation through further research and accumulation of additional safety data over time. 

## Conclusions

While the pharmacological profile of IN nalmefene has prompted concerns of an increased risk of precipitated withdrawal, post-marketing surveillance data collected to date have not substantiated these concerns. The availability and deployment of fast-acting, high-potency opioid receptor antagonists such as IN nalmefene are essential for improving clinical outcomes and reducing both morbidity and mortality amid an evolving opioid crisis currently driven by synthetic opioids. Ongoing research, robust surveillance, and comprehensive education efforts will be critical in assessing the prevalence of precipitated withdrawal following IN nalmefene and ensuring equitable access across the diverse populations impacted by the opioid crisis.
